# Prospective Surveillance and Molecular Characterization of Seasonal Influenza in a University Cohort in Singapore

**DOI:** 10.1371/journal.pone.0088345

**Published:** 2014-02-10

**Authors:** Ramandeep Kaur Virk, Paul Anantharajah Tambyah, Masafumi Inoue, Elizabeth Ai-Sim Lim, Ka-Wei Chan, Catherine Chua, Boon-Huan Tan

**Affiliations:** 1 Department of Medicine, National University of Singapore, Singapore, Singapore; 2 Experimental Therapeutics Centre, Agency for Science, Technology and Research, Singapore, Singapore; 3 Detection and Diagnostics Laboratory, DSO National Laboratories, Singapore, Singapore; 4 Department of Epidemiology and Public Health, National University of Singapore, Singapore, Singapore; 5 University Health Centre, National University of Singapore, Singapore, Singapore; Duke-NUS Gradute Medical School, Singapore

## Abstract

**Background:**

Southeast Asia is believed to be a potential locus for the emergence of novel influenza strains, and therefore accurate sentinel surveillance in the region is critical. Limited information exists on sentinel surveillance of influenza-like illness (ILI) in young adults in Singapore in a University campus setting. The objective of the present study was to determine the proportion of ILI caused by influenza A and B viruses in a university cohort in Singapore.

**Methodology/Principal Findings:**

We conducted a prospective surveillance study from May through October 2007, at the National University of Singapore (NUS). Basic demographic information and nasopharyngeal swabs were collected from students and staff with ILI. Reverse-transcriptase PCR (RT-PCR) and viral isolation were employed to detect influenza viruses. Sequencing of hemagglutinin (HA) and neuraminidase (NA) genes of some representative isolates was also performed. Overall proportions of influenza A and B virus infections were 47/266 (18%) and 9/266 (3%) respectively. The predominant subtype was A/H3N2 (55%) and the rest were A/H1N1 (45%). The overall sensitivity difference for detection of influenza A viruses using RT-PCR and viral isolation was 53%. Phylogenetic analyses of HA and NA gene sequences of Singapore strains showed identities higher than 98% within both the genes. The strains were more similar to strains included in the WHO vaccine recommendation for the following year (2008). Genetic markers of oseltamivir resistance were not detected in any of the sequenced Singapore isolates.

**Conclusions/Significance:**

HA and NA gene sequences of Singapore strains were similar to vaccine strains for the upcoming influenza season. No drug resistance was found. Sentinel surveillance on university campuses should make use of molecular methods to better detect emerging and re-emerging influenza viral threats.

## Introduction

Influenza virus is a major cause of morbidity and mortality worldwide. Influenza viruses are evolutionary dynamic viruses with high mutation rate [1]. Accurate detection and further subtyping of influenza A viruses is important for epidemiologic surveillance [2]. Many respiratory pathogens can present with “influenza-like” symptoms. Thus, infections caused by other respiratory pathogens may occasionally be difficult to distinguish from actual influenza infection on the basis of clinical features alone [3]. Therefore, accurate laboratory diagnosis is important in managing influenza virus infection. Most importantly, accurate laboratory diagnosis helps implement appropriate infection control strategies for individual as well as public health responses to further outbreaks [4], [5]. The superiority of molecular assays over conventional methods for diagnosis of respiratory viral infections in various populations is well established [6]–[8]. Viral isolation, however, provides an isolate of viable virus that can be used for comprehensive characterization of viruses.

Molecular characterization of circulating influenza A virus strains is essential for the selection of an optimal vaccine composition [2], to understand transmission characteristics and for monitoring drug resistance.

Neuraminidase inhibitors (NAIs), Oseltamivir and Zanamivir, interfere with the release of progeny viruses from the host cell and thus halt the spread of the virus [9]. The recent emergence of resistance to NAIs has necessitated a strong surveillance system to monitor resistance trends.

Influenza infection is a major cause of morbidity in young adults in Singapore with estimates of the economic impact of influenza including more than 3 million doctor visits and 2 million lost days of work [10]. Influenza in Singapore does not have well defined seasonality [11], [12] and tends to occur all year around. Relatively closed populations, such as, students living on campus, in dormitories or military personnel in camps have been proposed as sentinel sites for surveillance of novel influenza. The proportion of influenza-like illness (ILI) in young adults in Singapore due to actual influenza virus infection has only recently been defined in a military setting [13]. Military populations may not be the best for surveillance of ILI as they only interact within their localized community. University students, on the other hand, may be better than the military populations because local students reflect local community epidemiology as well as the many overseas students who may introduce new strains from their home country across the borders. In fact, in 1968, one of the best characterizations of the influenza pandemic was among students and staff of the then University of Singapore attending the University Health Centre (UHC) [14]. The potential for student health centre acting as sentinel surveillance site has not been thoroughly explored since then in the tropics and elsewhere.

We conducted a comprehensive prospective surveillance study in a university cohort to determine the proportion of ILI actually caused by influenza A and B viruses. The relative performances of reverse transcription-PCR (RT-PCR) and viral isolation for the detection of influenza A viruses were evaluated in parallel. We also carried out molecular characterization of some isolates to try to understand the molecular epidemiology of influenza in a semi-closed setting of a university by sequencing hemagglutinin (HA) and neuraminindase (NA) genes of influenza A/H3N2 and A/H1N1 viruses and by plotting phylogenetic trees. Genetic markers for resistance to Neuraminidase inhibitors (NAIs) were also investigated.

## Materials and Methods

### Study population

The majority of students and staff from the National University of Singapore (NUS) seek medical attention at the UHC. Individuals meeting the case definition for ILI of fever with respiratory symptom [15] were invited to participate in the study.

### Sample and Data Collection

After consenting, two nasopharyngeal swabs were collected from each participant by a trained research-assistant and placed in Copan’s Universal Transport media (Copan Diagnostics Inc., Murrieta, California). The samples were processed immediately or stored at –80°C until use. Basic demographic information was also collected.

### Ethics Statement

The study was approved by the NUS Institutional Review Board (IRB). The NUS-IRB reference number is 06-156 and approval number is NUS-282. Written informed consent was obtained from the participants before sample collection.

### Viral Isolation

Clinical samples (200 µl) were inoculated into 9 to 11-day-old embryonated chicken eggs [16] and incubated at 35°C for 3 days. Subsequently, the eggs were chilled at 4°C overnight or for 4 hours before harvesting. The allantoic fluid was harvested and inoculated into Madin-Darby Canine Kidney (MDCK, American Type Culture Collection ATCC, CCL-34, Rockville, MD, USA) cells grown on 12 mm coverslips. The coverslips were sterilized by dipping in 70% ethanol and flaming. Maintenance media comprising of Dulbecco’s Modified Eagle’s Medium (DMEM; Gibco BRL, Grand Island, NY, USA), L-1-tosylamido-2-phenylethyl chloromethyl ketone -Trypsin, bovine serum albumin, 100 U/ml of penicillin and 100 µg/ml of streptomycin was added [17]. The infection was carried out at 37°C in the presence of CO_2_. The cells were examined daily and harvested when extensive cytopathic effects (CPE) were observed. Seven days post-infection, the cells were fixed in 3% paraformaldehyde and processed for immunofluorescence assay.

### Immunofluorescent Antibody Staining (IFA)

The coverslips were treated with 0.1% saponin (Sigma, USA) and stained with monoclonal antibody against influenza A virus nucleoprotein antigen (Millipore, Bioscience Research Reagents, Temecula, CA) followed by fluorescein isothiocyanate-labeled goat anti-mouse immunoglobulin G (Millipore, Bioscience Research Reagents, Temecula, CA). The virus-infected cells were examined with a fluorescent microscope (OLYMPUS BX51). A scoring system was used for the intensity of bright apple green fluorescent nuclei. If more than 80% of the cells showed fluorescence, the slide was scored 3+; if 40–80% showed fluorescence, the slide was scored 2+; if 5–40% showed fluorescence, the slide was scored 1+; and if less than 5% showed fluorescence slide was considered negative.

### Multiplex End-point RT-PCR and Pyrosequencing

Total nucleic acids were extracted from viral transport media (VTM) samples using the RNeasy minikit (Qiagen, Inc., Valencia, CA, USA) following manufacturer’s instructions. Molecular diagnostics with primers targeting influenza A and B virus matrix (M) gene were performed, followed by a specific probe confirmation using Luminex xMAP-based assay (Luminex, Austin, TX, USA) as previously described [18]. The end-products were subjected to pyrosequencing, and the subtypes determined from the DNA sequences.

### Phylogenetic Analysis

Samples were randomly selected for sequencing from each month of the study period. Viral RNAs (vRNAs) were extracted from infected allantoic fluid of embryonated chicken eggs or VTM samples, using RNeasy mini kit (Qiagen Inc., Valencia, CA, USA) following manufacturer’s directions. Reverse Transcription (RT) of the vRNAs was performed with SuperScript First-Strand Synthesis System for RT-PCR (Invitrogen Corporation, CA, USA), using the Uni12 primer (5′AGCRAAAGCAGG3′) [18]. PCR amplification of full-length HA and NA genes was carried out using HiFi PlatTaq kit (Invitrogen) and primers as described by Hoffman et al [19]. The PCR products were purified using QIAquick Gel Extraction Kit (Qiagen Inc. Valencia, CA, USA) and sequenced with primers [19]–[21] listed in Table 1. Sequencing was performed with ABI Prism Big Dye Terminator (Applied Biosystems, Foster City, CA, USA). Raw sequence data were assembled and edited using SeqMan (DNASTAR, Lasergene Version 7, Madison USA). Nucleotide sequences of HA and NA genes were compared with each other, with vaccine strains and with other published 2007 sequences from GenBank (http://www-ncbi-nlm-nih-gov.libproxy1.nus.edu.sg/genomes/FLU/FLU.html) using Megalign (DNASTAR, Lasergene Version 7) by the Clustal W algorithm. For H3N2 subtype A/Winsconsin/67/2005 and A/Brisbane/10/2007 and for H1N1 subtype A/New Caledonia/20/1999, A/Solomon Islands/3/2006 and A/Brisbane/59/2007 were used. Percent (%) sequence homology was calculated for each of the full-length gene. Phylogenetic trees were constructed using the neighbor-joining method, with bootstrap analysis performed on 1000 replicates. Phylogenetic trees were viewed with TreeExplorer (v2.12,http://evolgen.biol.metro-u.ac.jp/TE/TE_man.html).

**Table 1:** Primer sets for sequencing HA and NA genes.

Serotype	Fragment	Forward Primer (5′- 3′)	Reverse Primer (5′- 3′)	Size (bp)
H3	F1	Bm-HA-1 ATTCGTCTAGGGAGCAAAAGCAGGGG	HA-R-504M13 CAGGAAACAGCTATGACCCATAGTCACGTTCAG	500
	F2	HA-F-391M13TGTAAAACGACGGCCAGTTATGCCTCCCTTAGG	HA-R-949M13CAGGAAACAGCTATGACCTCATTGGRAATGCTTC	580
	F3	HA-F-872M13TGTAAAACGACGGCCAGTAAGCTCRATAATGAG	Bm-NS-890ATATCGTCTCGTATTAGTAGAAACAAGGGTGTTTT	906
H1	F1	Bm-HA-1TATTCGTCTAGGGAGCAAAAGCAGGGG	HA1-6555CTACAGAGACATAAGCATTTC	650
	F2	HA1-490AATTTGCTATGGCTGACGGA	FluAHA1-1260CAATTTGTTGAATTCTTTGCCCACAG	770
	F3	FluAHA1-1180CCATTAATGGGATTACAAACAAGG	Bm-NS-890ATATCGTCTCGTATTAGTAGAAACAAGGGTGTTTT	600
N2	F1	Ba-NA-1TATTGGTCTCAGGGAGCAAAAGCAGGAGT	Na-R-560M13CAGGAAACAGCTATGACCTCGTGACAACTTGAGCTGGAC	560
	F2	Na_F_415M13TGTAAAACGACGGCCAGTTATCAATTTGCMCTTGGRCAGG	NA_R_984M13CAGGAAACAGCTATGACCAAGYCCTGAGCACACAT	567
	F3	NA_F_880BM13TGTAAAACGACGGCCAGTTCAGATGTRTHTGCM	Ba-NA-1413ATATGGTCTCGTATTAGTAGAAACAAGGAGTTTTTT	533
N1	F1	Ba-NA-1TATTGGTCTCAGGGAGCAAAAGCAGGAGT	FluNA1-550GCTGACCAAGCAACTGATTCAAAC	550
	F2	FluNA1-305CAGTGGGTGGGCTATATACACAAAAGA	Ba-NA-1413ATATGGTCTCGTATTAGTAGAAACAAGGAGTTTTTT	1100

*R =  A/G, Y =  C/T, M =  A/C, N =  A/C/G/T.

### Determination of influenza virus infection

PCR positivity was determined as previously described [18] and culture positivity was determined by scoring IFA results. Samples that tested positive by either RT-PCR or viral isolation or both, were regarded as true positive for influenza A virus infection. Only RT-PCR was used for testing influenza B virus.

### Statistical Analysis

Sensitivity, specificity, positive predictive value (PPV) and negative predictive value (NPV) were calculated using standard formulas.

### Nucleotide Sequence Accession Numbers

The sequenced Singapore isolates were deposited in GenBank and were assigned accession numbers from KF533050 to KF533066 and KF856946 to KF856951.

## Results

### General Findings

From May 2007 through October 2007, a total of 266 subjects participated in the study. There was no sample collection in June 2007 and July 2007 as the university was closed for vacations. One hundred and thirty-five (51%) males and one hundred and thirty-one (49%) females provided samples. The ages of the subjects ranged from 18 years to 60 years with a median of 23 years. Of the total of 266 subjects, 208 (78%) were students and 58/266 (22%) were staff. Amongst the 266 subjects, the proportion of Singaporeans was 146/266 and of non-Singaporeans was 120/266.

### Laboratory Analysis

**Figure 1:**
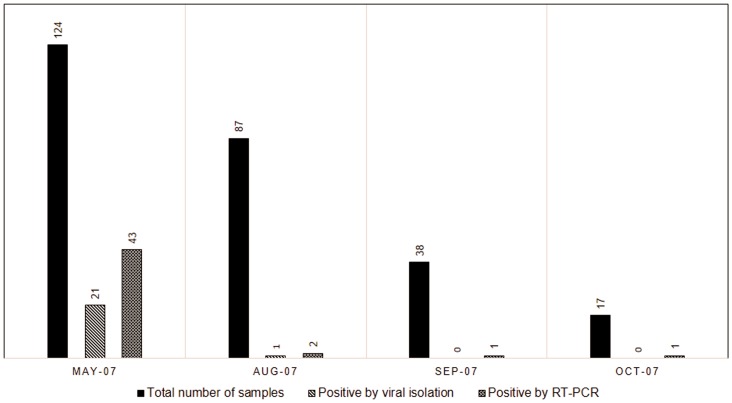
Monthly distribution of total number of samples and influenza A positive samples detected by RT-PCR and viral isolation during the study period.

Overall, 18% (47/266) of samples tested positive for the presence of influenza A virus and 3% (9/266) for influenza B virus. Eighteen percent of the samples were positive for influenza A virus by RT-PCR and 8% by viral isolation method (Table 2). In our study, 25 out of 47samples detected by RT-PCR were not detected by viral isolation but all samples positive by viral isolation were also positive by RT-PCR. Figure 1 shows the epidemiological curve describing influenza A virus infections detected employing RT-PCR and viral isolation. The peak in influenza A infection was observed in May. The predominant subtype was influenza A/H3N2 (55%) and the rest were A/H1N1 (45%).

**Table 2:** Number (%) of samples positive for influenza A virus infection by RT-PCR and viral isolation (shown in bold).

Diagnostic method	Influenza A virus infection	
	Present^1^	Absent^2^
Detected by RT-PCR	**47 (18%)**	0
Not detected by RT-PCR	0	219 (82%)
Detected by viral isolation	**22 (8%)**	0
Not detected by viral isolation	25 (9%)	219 (82%)

1 **Present** means sample was positive for influenza A infection by either or both the methods.

2 **Absent** means sample was negative for influenza A infection by both methods.

The overall sensitivity for RT-PCR was 100% (95% Confidence Interval; CI, 91–100%) whereas for viral isolation was 47% (95% CI, 32–62%).The sensitivity difference between RT-PCR and viral isolation method was 53%. The specificity and positive predictive value for each of the methods was 100%. The negative predictive values for RT-PCR and viral isolation were 100% and 90% respectively.

### Phylogenetic Analysis

Overall there were 22 sequences of influenza A virus successfully sequenced in this study (6 HA and 6 NA genes of A/H3N2 viruses; 5 HA and 5 NA genes of A/H1N1 viruses).

**Figure 2:**
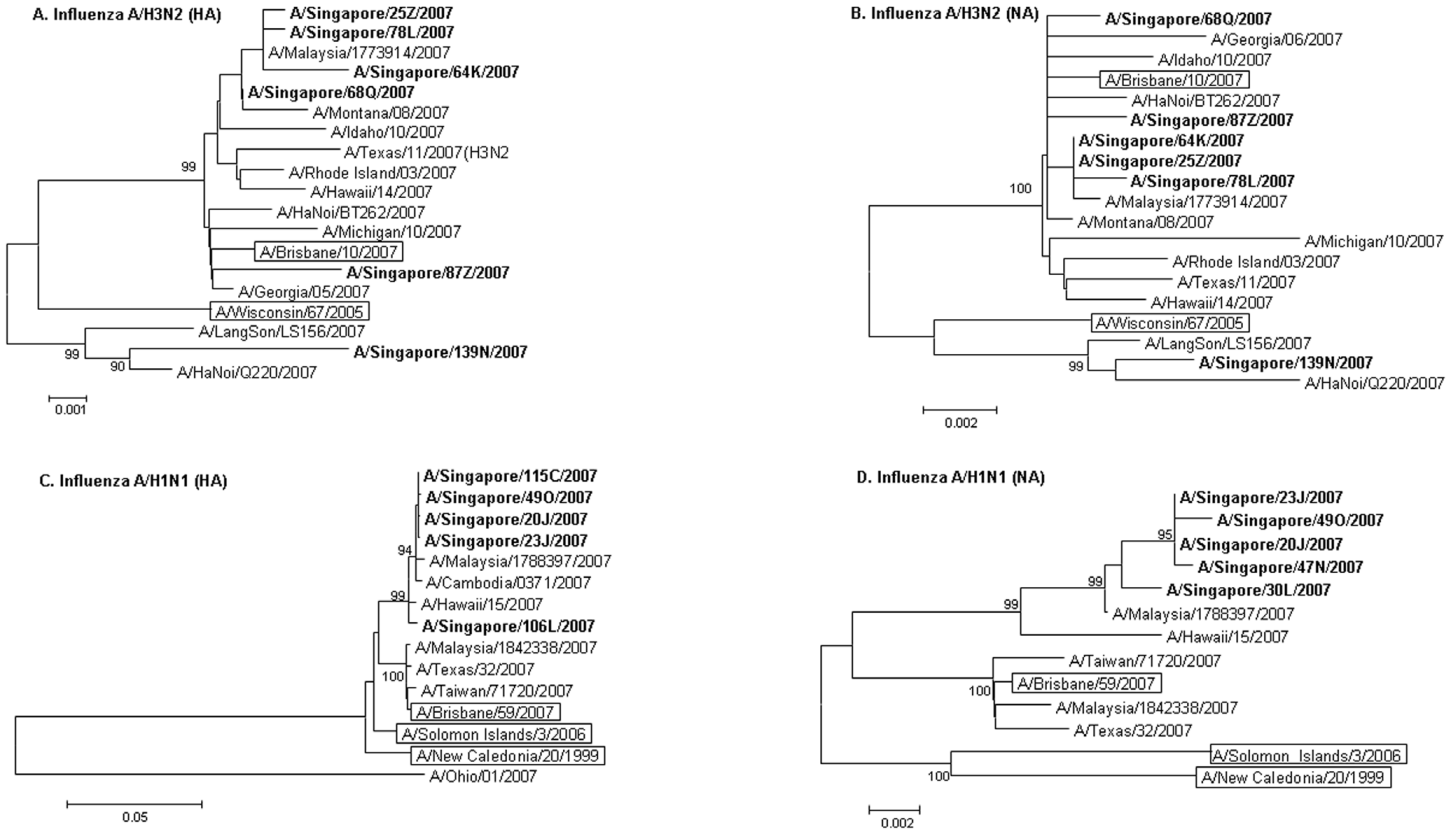
Phylogenetic trees of Seasonal influenza viruses circulating in a university cohort in Singapore, 2007. The phylogenetic trees of (A) 6 HA and (B) 6 (NA) genes of A/H3N2 viruses; (C) 5 HA and (D) 5 NA genes of A/H1N1 viruses with WHO vaccine strains and other 2007 sequences from GenBank constructed using neighbor-joining method. Bootstrap values 90 and over are shown. Singapore isolates are in bold and vaccine strains in rectangles.

**HA and NA diversity of seasonal influenza A/H3N2 viruses in Singapore, 2007.** The analysis of HA (Figure 2A) and NA gene (Figure 2B) of A/H3N2 viruses showed that the Singapore isolates shared >98% homology with vaccine strain of 2008-09 (A/Brisbane/10/2007). Percentage identity within the Singapore strains ranged from 98.20-99.88%. Notably, the genotype of the Singapore strains was different from the vaccine strain of 2007-08 (A/Winsconsin/67/2005) indicating antigenic drift.

**HA and NA diversity of seasonal influenza A/H1N1 viruses in Singapore, 2007.** The analysis of HA (Figure 2C) and NA genes (Figure 2D) of A/H1N1 viruses showed that the identity percentages within the genes sequences of Singapore strains ranged from 99.20-99.88%. Out of the three vaccine strains chosen for analysis, the Singapore strains showed greater similarity to vaccine strain of 2008-09 (A/Brisbane/59/2007).

### NAI Resistance

**Figure 3:**
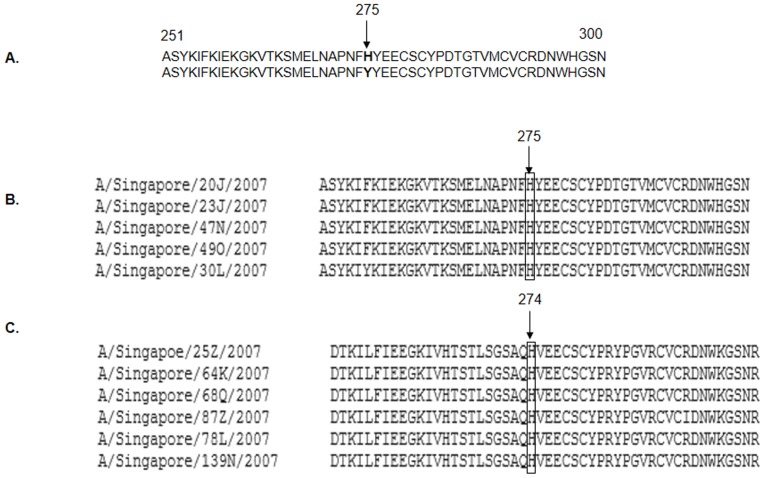
Influenza A Neuraminidase (NA) protein sequence Analysis for Oseltamivir Resistance. (A) The consensus sequence of neuraminidase (N1) gene from residues 251 to 300. Substitution of amino acid Histidine (H) to Tyrosine (Y) at position 275 in N1 gene (shown in bold) and at position 274 in N2 gene confers resistance to oseltamivir. Protein sequences of (B) N1 and (C) N2 genes of Singapore isolates.

Deduced aminoacid sequences of NA genes of the Singapore strains were screened using MultAlin [22] for the genetic markers of oseltamivir resistance. These were H274Y/H275Y mutation and other substitution mutations like A/H3N2 (E41G, E119V/G/D, Q136K, D151A/V/N, R152K, V165I, I222R/Q, Q226H, G248R, K249E, D251G, H274N, R292K, and N294S) and A/H1N1 (D79G, H126N, Q136K, Y155H, S247G, G248R, and I266V). The sequenced Singapore strains did not harbor any of the genetic markers of resistance to oseltamivir (Figure 3).

## Discussion

ILI surveillance is important for influenza preparedness plans globally. While there is huge body of literature on the proportion of ILI due to actual influenza infection in temperate regions [23], [24], its knowledge remains limited in the tropics. There are only a few studies on university students in tropical and subtropical settings. There is a study that assessed ILI in university students presenting to university health clinic in Florida, USA [23]. The study, however, was done on a small cohort of 60 participants and influenza infection was confirmed in 63% of participants. Another study with same number of participants was conducted in 2002 in San Francisco [24] and influenza was detected in 20% of students, which is similar to our findings.

In our prospective study of 266 students and staff with ILI, we found an 18% positive rate for influenza A virus. This is slightly lower than the positive rate for influenza A virus of 24% found in a military study in Singapore using molecular-based diagnostics [13]. The peak in influenza A infection was observed in May. May is the traditional influenza peak season in Singapore [11]. It is also the examination season in the NUS and students are perceived to be at a higher risk of upper respiratory tract infections. In general, we found RT-PCR superior than viral isolation. The lower sensitivity of viral isolation may be attributable to inactivation of virus during transportation to laboratory [25]. Overall, there was a 53% sensitivity difference between RT-PCR and viral isolation in detecting influenza A virus. A sensitivity difference of 9–40% for detection of seasonal influenza A virus has been reported [9], [26]–[29]. We found a slightly wider sensitivity gap of 53% for seasonal influenza A probably because of the difficulty in culturing the circulating A/H3N2 strains. This may be attributable to different replication capacity of each strain of influenza A virus during viral isolation. RT-PCR on the other hand is not affected by variations in growth characteristics. The predominant subtype was A/H3N2 and this finding is in agreement with another study done in Singapore [30] and with the national surveillance data published by the Ministry of Health, Singapore [31].

The phylogenetic analyses shows that, both the HA and the NA genes of sequenced influenza A/H3N2 and A/H1N1 viruses clustered with WHO recommended vaccine strains of the upcoming influenza season. This suggests that surveillance of influenza viruses is essential for optimal vaccine composition. Furthermore, majority of the Singapore strains that we sequenced were closely related to each other by sequence analysis, this suggests that the majority of influenza was localized. This has implications for the response to future pandemics. In such cases, closure of large institutions may be an important and useful strategy. The data are limited, however, and further research is needed to validate this. During the 2007–2008 influenza season, oseltamivir resistance among influenza A /H1N1 viruses increased significantly for the first time worldwide [32]. Genetic markers of oseltamivir resistance, however, were not detected in the sequenced Singapore isolates.

Our study has a few limitations. We collected data from a single university so our results cannot be generalized to the general population. Also, we did not test for other respiratory pathogens. Our analyses were conducted on only 22 sequences and this highlights that sequencing a few isolates is not sufficient to capture the epidemiology of cohort studies. Nevertheless, since a limited amount of research exists on ILI surveillance in young adults in Singapore, to our knowledge only one study in a military population has been published and none in a college setting in recent years, our study provides the baseline surveillance data for the proportion of influenza viruses in young adults with ILI in a university setting. We were also able to identify strains that were closely related to strains that subsequently became dominant globally among those circulating in staff and students at our university.

## Conclusions

Populations such as university campuses are useful sentinel sites and should be routinely monitored together with military campuses, childcare centers and other similar cohorts. Surveillance and control of influenza in large tropical institutions may be an important and useful strategy in containing the next pandemic. Our results also show that seasonal influenza A is more readily detected by molecular-based than by viral isolation methods. This further suggests that sentinel surveillance should make use of molecular-based methods to better detect emerging and re-emerging influenza viral threats.
